# Sensitivity enhancement of DHR123 radio-fluorogenic nanoclay gel dosimeter by incorporating surfactants and halogenides†

**DOI:** 10.1039/d0ra02717k

**Published:** 2020-08-04

**Authors:** Anri Mochizuki, Takuya Maeyama, Yusuke Watanabe, Shinya Mizukami

**Affiliations:** Department of Chemistry, School of Science, Kitasato University 1-15-1 Kitasato, Minami Sagamihara Kanagawa 252-0373 Japan maeyama@kitasato-u.ac.jp; School of Allied Health Sciences, Kitasato University 1-15-1 Kitasato, Minami Sagamihara Kanagawa 252-0373 Japan

## Abstract

Dosimetry of spatial dose distribution of ionizing radiation in tissue equivalent materials is particularly important for cancer radiotherapy. Here, we describe a radio-fluorogenic gel-based dosimeter that has achieved 16 times higher sensitivity by incorporating surfactants and halogenides. The gel dosimeters were prepared from dihydrorhodamine 123 (DHR123) and small amounts of nano-sized clay and a radiosensitizer. By comprehensively changing the type of additives for the sensitizer (three surfactants: Triton X-100, sodium dodecyl sulfate (SDS) and cetyltrimethylammonium bromide, and three halogenides: trichloroacetic acid, tribromoacetic acid and 2,2,2-trichloroethanol), the increase in sensitivity can be explained by an increase in relative fluorescence quantum yield and an increase in radiation chemical yield. These highly sensitive gel dosimeters also show dose rate independent sensitivity under irradiation at 0.64 and 0.77 Gy min^−1^ using a 6 MV X-ray therapeutic beam from the medical linac.

## Abstract 2

Dosimetry of spatial dose distribution of ionizing radiation in tissue equivalent materials using high sensitive radio-fluorogenic gel dosimeter using DHR123 with sensitizer. (Radiation therapy planning image courtesy of Varian Medical Systems, Inc. All rights reserved.)

## Graphical abstract

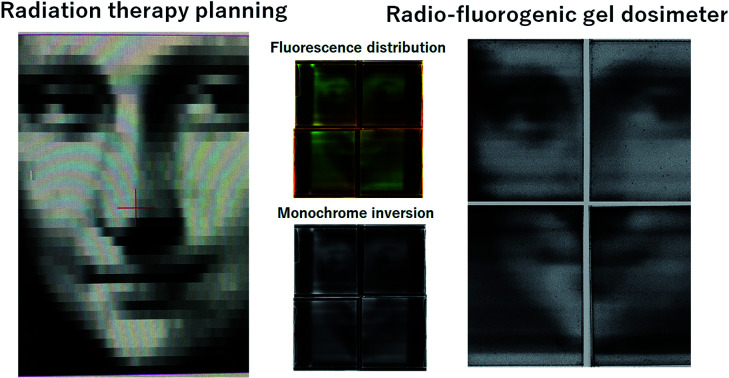

## Introduction

In cancer radiotherapy, a three-dimensional (3D) dose distribution is adjusted to the shape of a tumor. High-precision radiotherapy that focuses high doses of radiation on tumors and decreases the amount of damage to healthy tissues is performed. To validate the complex dose distribution, a dosimetry is required that is tissue equivalent and has high spatial resolution in three dimensions.^1–3^

Dihydrorhodamine 123 (DHR123) has been used as one of the sensitive probes in the production of reactive oxidizing species (ROS) in cells.^4^ Non-fluorescent DHR123s are converted to highly fluorescent forms, rhodamine 123 (RD123) in the presence of ROS, such as OH radical; hence, the fluorescence intensity is proportional to the amount of reacted ROS. Recently, the DHR123 probe was applied to ionizing radiation dosimeter,^5^ since this DHR123 probe also work for ionizing radiation induced ROS.^6^ The fluorescence intensity increases with the increase in the absorbed dose. While the fluorescence distribution corresponding to dose distribution was maintained in the hydro nano-clay gel matrix, DHR123 radio-fluorogenic nano-clay gel (DHR123RFG) dosimeter produced two-dimensional (2D) dosimetry with a 2D fluorescent scanner. This hydrogel dosimeter prepared mainly with water and a small amount of gelling materials that is tissue equivalent material is expected to be used as a verification method of dose distribution in radiotherapy.

As a typical gel dosimeter,^7^ there are Fricke gel^8–11^ and polymer gel^12–15^ dosimeters, and many radiosensitizers^2,16–20^ have been investigated because improving sensitivity is an issue, up to now. On the other hand, there are not many reports in the gel dosimeters utilizing fluorometry using radiosensitizers, although there are several type of radio-fluorogenic gels.^21–27^ Thus, the purpose of this study is investigation of sensitizer effects on DHR123RFG in detail.

The reaction mechanism of the DHR123RFG^6^ is thought to be the same as the radio-chromic hydrogels using leuco dye such as leuco crystal violet and leuco malachite green,^28,29^ although the DHR123RFG is a specific gel dosimeter which uses the fluorescence characteristics. In the radio-chromic hydrogels, the sensitivity enhancement is reported by the addition of a surfactant and a halide. The surfactant in these phantoms helps to solubilize the leuco-dye molecules, which are only sparingly soluble in water.^28^ The halide increases the efficiency of product yield.^30^ The dose rate dependence of these addition effects was also discussed.^31^ In this study, we apply sensitizers to the DHR123RFG based on the abovementioned research. Its sensitivity characteristics and dose rate dependence are evaluated, and the mechanism of the sensitization effect also is discussed.

## Experimental

**Fig. 1:**
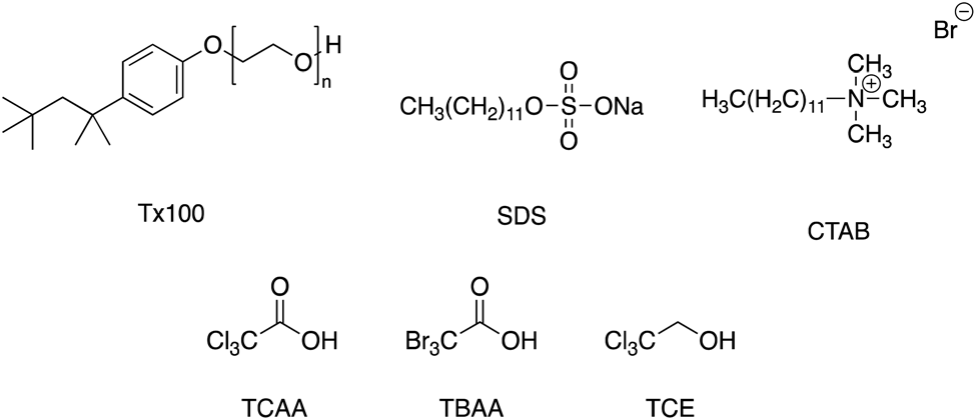
Chemical structure of surfactants and halogenide.

In the sample preparation, we used three different types of surfactants; a non-ionic surfactant (Triton X-100 (Tx100)), an anionic surfactant (sodium dodecyl sulfate (SDS)), and a cationic surfactant (cetyl trimethyl ammonium bromide (CTAB)), and three halogenide namely; trichloroacetic acid (TCAA), tribromoacetic acid (TBAA), and 2,2,2-trichloroethanol (TCE) as shown in Fig. 1. The prepared conditions were summarized in Table 1, 2, and 3 for surfactant-dependent, halogenide-dependent, or both respectively. Detailed preparation procedures are described in our previous report.^5^

**Table 1:** Prepared conditions of gels made with surfactant

No.	Surfactant	Conc. [mM]	DHR123 [μM]	Nanoclay [wt%]
1			100	2.5
2.1	Tx100	0.25	100	2.5
2.2	Tx100	0.5	100	2.5
2.3	Tx100	1	100	2.5
2.4	Tx100	4	100	2.5
2.5	Tx100	38	100	2.5
2.6	SDS	0.25	100	2.5
2.7	SDS	0.5	100	2.5
2.8	SDS	1	100	2.5
2.9	SDS	2.5	100	2.5
2.10	SDS	17	100	2.5
2.11	CTAB	0.5	100	2.5

**Table 2:** Prepared conditions of gels made with halogenide

No.	Halogenide	Conc. [mM]	DHR123 [μM]	Nanoclay [wt%]
1			100	2.5
3.1	TCAA	0.25	100	2.5
3.2	TCAA	0.5	100	2.5
3.3	TCAA	1	100	2.5
3.4	TCAA	5	100	2.5
3.5	TBAA	0.25	100	2.5
3.6	TBAA	0.5	100	2.5
3.7	TBAA	1	100	2.5
3.8	TBAA	5	100	2.5
3.9	TCE	0.25	100	2.5
3.10	TCE	0.5	100	2.5
3.11	TCE	1	100	2.5
3.12	TCE	5	100	2.5
3.13	TCE	50	100	2.5

**Table 3:** Prepared conditions of 100 μM DHR123 and 2.5 wt% nanoclay gels made with surfactant and halogenide and irradiation condition

No.	Surfactant	Conc. [mM]	Halogenide	Conc. [mM]	Dose rate [Gy min^−1^]
1					4.64 or 0.77
4.1	Tx100	38			
4.2	Tx100	38	TBAA	0.5	
4.3	Tx100	38	TBAA	1	
4.4	SDS	17			
4.5	SDS	17	TBAA	0.5	
4.6	SDS	17	TBAA	1	

Gel dosimeter irradiation was performed using a 6 MV X-ray beam generated with a Varian Linear Accelerator (TrueBeam, Varian Medical Systems, Palo Alto, USA). The radiation dose ranges for all the prepared samples are 0–4 Gy. A dose rate of 4.66 Gy min^−1^ was used for samples shown in Tables 1 and 2, while two dose rates (4.66 and 0.77 Gy min^−1^) were used for samples shown in Table 3. Within one week of irradiation, fluorescence measurements of the irradiated samples were conducted using an F-4500 spectrofluorometer (Hitachi, Japan). The exciting wavelength is 480 nm and the emission and excitation slit widths were set at 1 nm and 20 nm, respectively, and these parameters were kept constant for the measurements of all samples.

The yield of RD123 [*G*(RD123)] was calculated by using the formula (1) wherein Δ*I* is the increasing rate of fluorescence intensity per 1 Gy and *Φ*_f_ is the relative fluorescence quantum yield of RD123 calculated from the slope of the calibration curve (No. 5.1 in Table 4). The effects of the sensitizer on relative fluorescence quantum yield of RD123 also evaluate under several condition as shown in Table 4 (No. 5.2 to 5.9).1
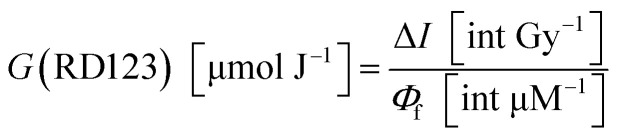


**Table 4:** Prepared conditions of the calibration curve

No.	Additive	Conc. [mM]	RD123 [μM]	DHR123 [μM]
5.1			0.026–0.21	100
			0.25–2.1	
5.2	TBAA	1	0.21	100
5.3	Tx100	0.95	0.21	100
5.4	Tx100	3.8	0.21	100
5.5	Tx100	38	0.21	100
5.6	SDS	1	0.21	100
5.7	SDS	2.6	0.21	100
5.8	SDS	17	0.21	100
5.9			0.026–0.21	0
			0.25–2.1	

## Results and discussion

### Effect of different surfactants and surfactant concentrations on dose sensitivity

We evaluated the sensitivity of the DHR123RFG dosimeter by linear fitting (Fig. S1–S8:† the dose dependence of changes in the fluorescence intensity). In all cases, good linearity was observed under identical fluorescence measurement conditions within the dose region of 0–4 Gy except for CTAB (No. 2.11). Because the gels made with CTAB (No. 2.11) became clouded, we did not irradiate. It is considered that CTAB clump together between clay layers and did not disperse because CTAB is a cationic surfactant.

**Fig. 2:**
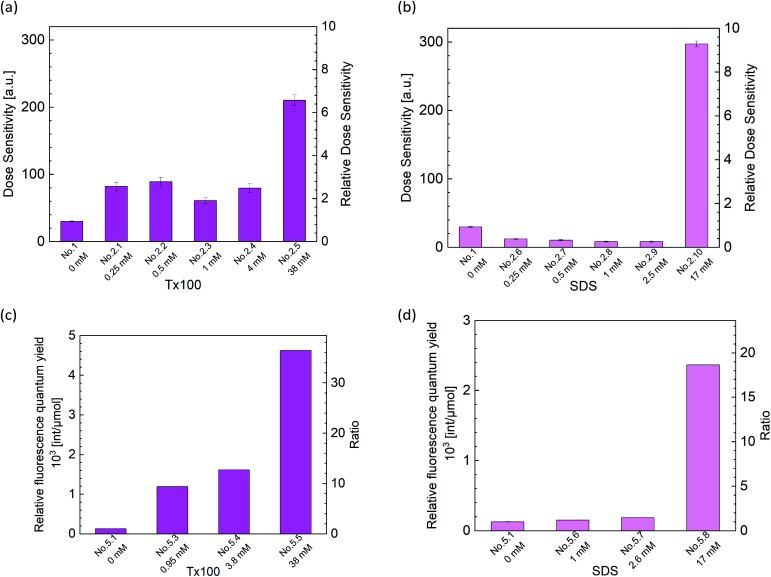
(a) and (b) Effect of surfactant concentrations on dose sensitivity ((a) Tx100, (b) SDS) (c) and (d) effect of surfactant concentrations on the relative fluorescence quantum yield ((c) Tx100, (d) SDS).

Fig. 2(a) and (b) show the effect of surfactant concentrations of Tx100 and SDS on dose sensitivity, respectively. The values of dose sensitivity with standard error, its ratio, and initial fluorescence intensity are also listed in Tables S1–S3 in the ESI.†Fig. 2(c) and (d) shows the effect of surfactant concentrations on relative fluorescence quantum yield of RD123 calculated from the calibration curve which added surfactants. The right-hand axis shows the ratio which is normalized by the slope of standard calibration curve (No. 5.1 in Table 4).

From these results, it was found that the sensitivity was increased by up to about 2.5 times by Tx100 (0.25–4 mM), and by further addition of Tx100 (38 mM), the sensitivity showed 7 times higher than the standard sample. It was also found that the relative fluorescence quantum yield of the RD123 increased with the increase in Tx100 concentration. This is a reasonable explanation for sensitivity enhancement. In other words, the RD123 which is produced by the radiation-induced reactions of the DHR123RFG shows stronger fluorescence intensity by the presence of Tx100, and the sensitivity also increased. When 0.25 to 2.5 mM of SDS was added, there was no increase in the sensitivity (gel 2.6 to 2.9 in Table 1); however, on the addition of 17 mM SDS, the sensitivity was found to increase by 8 times (gel 2.10 in Table 1). Unlike in Tx100, from Fig. 2(d), the relative fluorescence quantum yield of RD123 only got higher by adding 17 mM SDS, while other samples (gel 5.6, 5.7 in Table 4) one did not change, and it agrees with above dose sensitivity results. In generally, it is considered that the relative fluorescence quantum yield changes due to two factors, the aggregation effects of the fluorescence dyes and the solvent effect. In the former, the surfactant prevents the aggregation of dyes, and increase the quantum yield of RD123 as reported in the literature using another fluorescence dye.^32,33^ In the latter case, the surfactant increases the relative fluorescence quantum yield by giving the different environment than simple aqueous solution such as micelles.^34^ These phenomena show the different degrees of effect depending on the dye, but an increase in quantum yield of fluorescent dyes using surfactants and clay has been reported.^35^

**Fig. 3:**
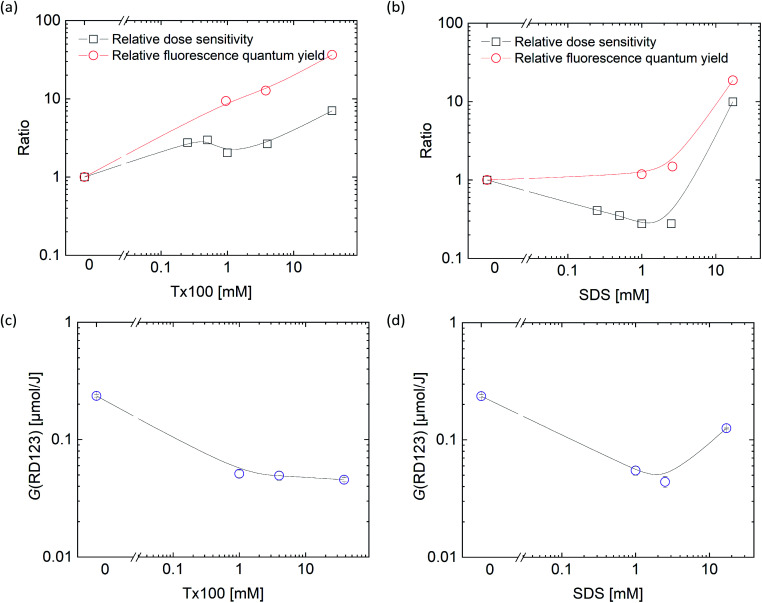
Surfactant concentration dependence on the relative fluorescence quantum yield and the relative dose sensitivity ((a) Tx100, (b) SDS), and on the yield, *G*(RD123) ((c) Tx100, (d) SDS).

To confirm the sensitizing effects of surfactants in detail, we compared the surfactant concentration dependence of (black square) and relative fluorescence quantum yield (red square) which normalized the value of standard sample (Fig. 3). Relative fluorescence quantum yield is higher than relative dose sensitivity in both surfactants. Thus, it was suggested that radiation chemical yields decreased by addition of surfactants.

To discuss in more detail, the effects of surfactant concentrations on *G*(RD123) [μmol J^−1^] were shown in Fig. 3. *G*(RD123) calculated using formula (1). On both surfactants, *G*(RD123) was reduced to one-sixth by the addition of 0.25 mM; therefore, it is considered that the surfactants that inhabit reaction of DHR123 with hydroxyl radicals, gets the final production efficiency of RD123 lower. Although, for the Tx100, *G*(RD123) did not depend on surfactant concentration in a range 1–38 mM, for the SDS, *G*(RD123) made with 17 mM increases to 0.125 μmol J^−1^. This observation showed that a high concentration of SDS did not inhibit the reaction of DHR123 with water decomposition radicals and it effectively increased the relative fluorescence quantum yield of RD123. On the addition of 17 mM SDS, the relative fluorescence quantum yield increased rapidly; hence, the higher dose sensitivity is attributed to the process that involves the interaction between DHR123RFG and surfactant. It was an interesting result because, in LCV gel dosimeters, the effect of the presence or the absence of surfactant on dose sensitivity was not discussed due to the low solubilities of LCV.^31^

**Fig. 4:**
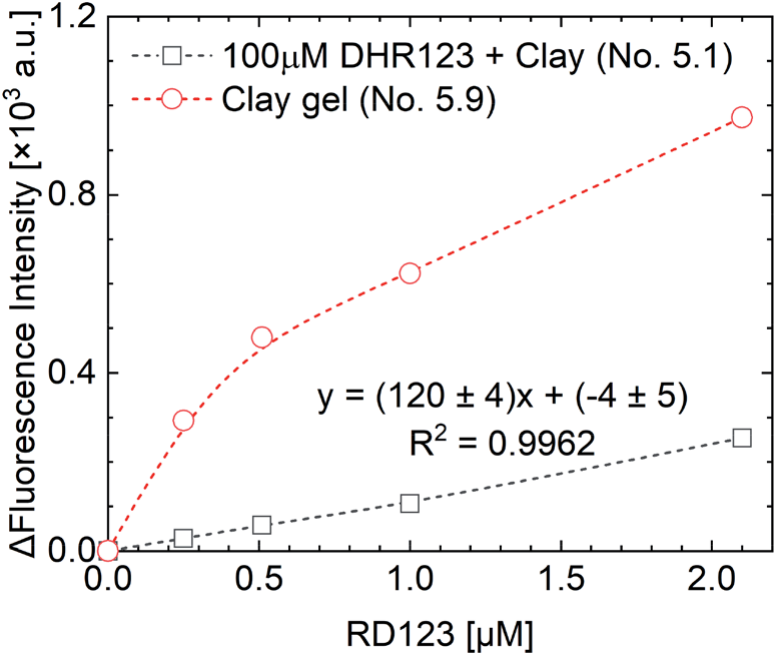
Fluorescence intensity as a function of RD123 concentration for calibration curve.

**Fig. 5:**
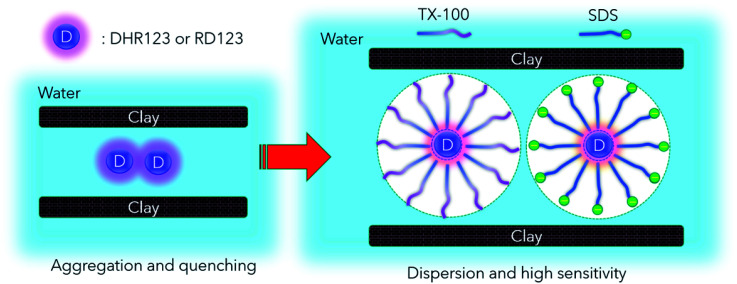
Main mechanism of sensitivity increase by adding surfactant.

The measurement results of the calibration curve with and without non-fluorescent DHR123 are shown in Fig. 4 (comparison between No. 5.1 and No. 5.9 in Table 4). The fluorescence intensity of RD123 varied greatly depending on the presence or absence of DHR123, which revealed that DHR123 acts as a quencher of RD123. Since the DHR123 gel dosimeter after irradiation also include an excess of DHR123 (100 μM order) with respect to RD123 (several μM order), it is considered that the quenching effect of DHR123 on the sensitivity of the gel dosimeter is large. In other words, as shown in the Fig. 5, a nonfluorescent uncharged compound, DHR123, has a low solubility in water, so the two surfactants become efficient DHR123 dispersants, thereby reducing their role as quenchers. And it is speculated that an increase in sensitivity has occurred. Clay-dispersed hydrogel and water cannot be considered as the same solvent, but SDS and Tx-100 do not contribute to the improvement of the quantum yield of RD123 alone in the studies of investigating the influence of the surfactant of DHR123 in the aqueous solution.^36^ Therefore, it is also possibly caused that the dye aggregates due to the presence of clay.

Next, comparing Tx100 and SDS, SDS contains Na^+^ counter ions. However, this Na^+^ ion has low reactivity with water decomposed radicals,^37^ and is considered not to participate in the reaction of DHR123. Nonionic Tx100 and anionic SDS have different micellar structures. It has also been pointed out that SDS, which produces highly polar micelles, has a higher H_3_O^+^ concentration.^38^ It is presumed that there is a good influence on the reaction yield or fluorescence quantum yield due to the change in pH. It is necessary to control each component of RD123 and DHR123, and to investigate the effect of adding a surfactant for further consideration. Moreover, when these two surfactants have different dispersion effects, it is supposed that adding both surfactants at the same time is also effective.^38,39^

### Effect of different halogenides and halogenide concentrations on dose sensitivity

**Fig. 6:**
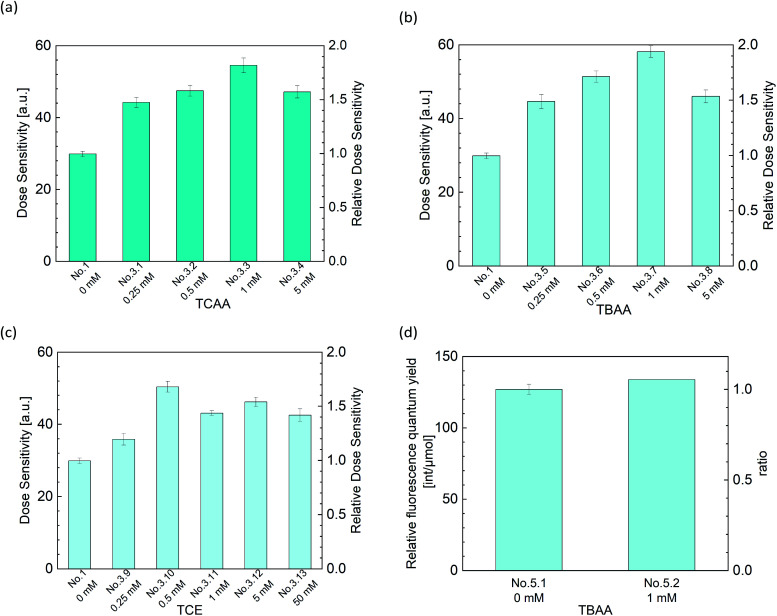
(a)–(c) Effect of halogenide concentrations on dose sensitivity ((a) TCAA, (b) TBAA and (c) TCE) (d) effect of TBAA concentrations on the fluorescence quantum yield.

**Fig. 7:**
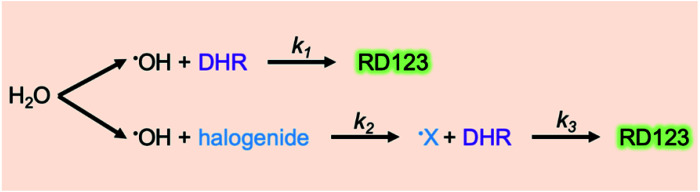
The production reaction of RD123 in DHR123 fluorescent gel dosimeter.

From Fig. 6(a)–(c), we can see that three types of halogenide, TCAA, TBAA, TCE worked as a sensitizer of the DHR123RFG. Gels became clouded when added more than 5 mM TCAA or TBAA and it was hard to experiments. In contrast, in terms of TCE, the gel was clear when added even 50 mM. On the other hand, it has been found that the addition of excess halogenide didn't contribute to the increase in sensitivity. The sensitivity got to the highest when added 1 mM TCAA, TBAA or 0.5 mM TCE. The maximum values of increasing rate were respectively 1.82, 1.95, and 1.69. We evaluated the relative fluorescence quantum yield when added halogenides to examine the mechanism of increasing sensitivity in the same way as in the previous section. Fig. 6(d) shows the effect of TBAA on relative fluorescence quantum yield (in the left-hand axis) and the relative value which normalized the relative fluorescence quantum yield of the standard sample (No. 1) (in the right-hand axis). The relative fluorescence quantum yield did not change by the addition of TBAA. We confirmed that TCAA also did not change the relative fluorescence quantum yield (not shown in this paper). It is considered that gels made with halogenide do not have a sensitizing effect of increasing the relative fluorescence quantum yield. In general, halogenides are known to increase the sensitivity through two mechanisms,^40,41^ one is through an increase in the acidity and the other is through the formation of halogen radical (˙X). Because the pH of gels did not change in present conditions, the former did not contribute to the increasing the sensitivity. Therefore, it is thought that the main mechanism for the increase in sensitivity in the DHR123RFG is the formation of halogen radical. It is known that the hydroxyl radicals show high reactivity but least efficiency in generating RD123 since hydroxyl radicals show lower selectivity and attack various parts of DHR123 (ref. 6). Because the redox potential of the Cl˙ radical and Br˙ radicals are lower than that of hydroxyl radical,^42^ these halogen radicals show higher selectivity than hydroxyl radicals and better efficiency in generating RD123. According to the above discussion, the main reaction of the DHR123 fluorescent gel dosimeter made with halogenide involves three reactions shown in Fig. 7.

(1) The hydroxyl radical scavenging reaction by DHR123 (*k*_1_), (2) the hydroxyl radical scavenging reaction by halogenide (*k*_2_), (3) the oxidation reaction of DHR123 by halogen radical (*k*_3_) produced by the second reaction. It is considered that the higher the concentration of halogenide, the faster the rate of the second reaction (*k*_2_) than that of the first reaction (*k*_1_) and the production quantity of halogen radical gets larger. Therefore, the oxidation reaction of DHR123 occurs more easily and the production quantity of RD123 gets larger. However, in the case of adding excess halogenide, it is presumed that other minor reactions, such as the reaction of halogenides occur against the reaction of DHR123 with a halogen radical (*k*_3_). As a result, the production quantity of RD123 decreased a little. Considering each rate constant, for example, in terms of TCE, the reactivity with hydroxyl radical is reported. The following showed the rate constants of DHR123 (ref. 6) and TCE^37^ with hydroxyl radical in eqn (2) and (3) respectively.

2 DHR123 + ˙OH → DHR–OH˙, *k*_1_ = (3.2 ± 0.1) × 10^10^ M^−1^ s^−1^

3 CCl_3_CH_2_OH + ˙OH → product, *k*_2_ = 4.2 × 10^8^ M^−1^ s^−1^

Comparing these two rate constants, *k*_1_ is 100 times larger than *k*_2_, the ratio of scavenging hydroxyl radical by TCE (0.5 mM) is 0.06%. It showed a low value and the reaction of TCE with hydroxyl radical rarely occurs. On the other hand, experimental results show the sensitivity enhancement by addition of TCE. Thus, we considered that the rate constant of DHR123 with hydroxyl radical is about 10^8^ M^−1^ s^−1^, since RD123 which had a similar structure with DHR123 with hydroxyl radical reported 1.6 × 10^8^ M^−1^ s^−1^.^6^ In this case, the ratio of the scavenging hydroxyl radical calculated with TCE (0.5 mM) using the rate constant *k*_1_ = 10^8^ M^−1^ s^−1^ is 95%, therefore DHR123 can react with only 5% of the hydroxyl radical. This rate constant is thought to be appropriate because the dose sensitivity did not change despite adding more than 0.5 mM TCE.

### Effect of both surfactants and halogenides on dose sensitivity

**Fig. 8:**
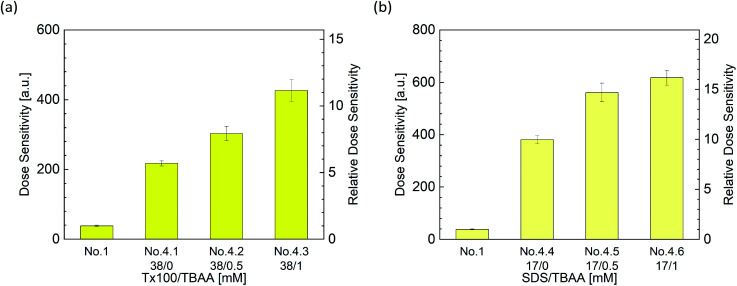
Effect of surfactant and halogenide, TBAA concentrations on dose sensitivity ((a) Tx100, (b) SDS).

Fig. 8 show the dose sensitivity of DHR123RFG dosimeter made with surfactant and halogenide (TBAA) and the relative sensitivity of it normalized by standard sample (No. 1). Gels made with both surfactant and halogenide show higher sensitivity than gels made with surfactant only (No. 2.5 and 4.1). It can be observed that the gel made with Tx100 and TBAA which showed maximum sensitivity showed 11 times higher than the standard sample (No. 1) while the gel made with SDS and TBAA which showed maximum sensitivity showed 16 times higher than the standard sample (No. 1).

The fluorescence intensity, *I*_f_, can be expressed by the formula (4) wherein *ε* is a molar extinction coefficient, *c* is a concentration of the fluorescent substance, *d* is an optical path-length (1 cm), *I*_0_ is an intensity of excitation light, and *Φ*_f_ is a relative fluorescence quantum yield.

4 *I*_f_ ∝ *ε* × *c* × *d* × *I*_0_ × *Φ*_f_

It is considered that the anticipated increasing rate of sensitivity, Δ*I*_f_, can be expressed by formula (5) wherein Δ*c* is the increasing rate of the production concentration of RD123 by adding halogenide and Δ*Φ*_f_ is the increasing rate of the relative fluorescence quantum yield of RD123 (considering inhabitation of reaction by surfactant) by adding surfactant.

5 Δ*I*_f_ ∝ Δ*c* × Δ*Φ*_f_

The increasing ratio of dose sensitivity by adding halogenide (TBAA) is 1.72 (±0.05) in 0.5 mM and 1.95 (±0.05) in 1 mM (Table S2†); the increasing ratio of dose sensitivity by adding surfactant is 6.36 (±0.45) in 38 mM Tx100 (average of relative dose sensitivity of No. 2.5 and No. 4.1 in Tables S1 and S3†) and 9.96 (±0.29) in 17 mM SDS (average of relative dose sensitivity of No. 2.10 and No. 4.4). From the calculated anticipated Δ*I*_f_ using these values and the formula (5), the increasing ratio is 1.72 × 6.36 = 10.9 in 0.5 mM TBAA and 12.4 in 1 mM TBAA. Comparing the measured value, 7.93 ± 0.55 in 0.5 mM TBAA (No. 4.2), 11.2 ± 0.8 in 1 mM TBAA (No. 4.3), and the calculated value, the calculated value was close to the measured value.

In the case of SDS, the anticipated Δ*I*_f_ which was calculated using the formula (5) like in Tx100, the increasing ratio is 17.1 in 0.5 mM TBAA and 19.4 in 1 mM TBAA. Comparing the measured value, 14.7 ± 1.0 in 0.5 mM TBAA (No. 4.5), 16.2 ± 0.8 in 1 mM TBAA (No. 4.6), and the calculated value, the calculated value was close to the measured value, as well as in Tx100. Therefore, the effect of surfactants and halogenides on the dose sensitivity can be expressed by the formula (5), and it showed that the contributions of the surfactant and the halogenide did not interfere with each other.

### Dose rate dependence of DHR123RFG prepared by improved conditions

Table 5 shows the dose rate dependence of the sensitivity of DHR123RFG prepared by improved conditions. The relative sensitivity is normalized by the sensitivity at 4.66 Gy min^−1^ in each condition. The standard error of dose sensitivity of all samples was under 8%, it was thought that there is dose rate dependence if the difference between the sensitivity at 4.64 Gy min^−1^ and 0.77 Gy min^−1^ is more than 10%. In gels made with Tx100, gel made without TBAA (No. 4.1) and with 0.5 mM TBAA (No. 4.2) showed dose rate dependence, however, a gel made with 1 mM TBAA (No. 4.3) did not show dose rate dependence. It was found that the increase in the concentration of TBAA improves the dose rate dependence. This phenomenon also described in the report that the dose rate dependence of LCV gel dosimeter is improved by increasing the concentration of TCE.^43^ In gels made with SDS, all gels did not show dose rate dependence regardless of addition of TBAA. In conclusion, DHR123RFG dosimeter made with 1 mM TBAA and 38 mM Tx100 (No. 4.3) and made with 1 mM TBAA and 17 mM SDS (No. 4.6) showed approximately 10 or more times higher sensitivity than standard sample (No. 1), and gels made under these conditions can measure the dose even from 0.01 Gy because the detection limit of the standard sample is 0.1 Gy and with dose rate independence radiological properties.

**Table 5:** Influence of Tx100, TBAA, and the both, on the dose sensitivity in different dose rates

No.	Surfactant and additive		Dose rate [Gy min^−1^]	Dose sensitivity [a.u.]	Ratio
1	0	0	4.64	38.23 ± 1.78	1.02
			0.77	38.94 ± 2.27	
4.1	38 mM Tx100	0	4.64	217.1 ± 7.15	1.15
			0.77	250.5 ± 11.65	
4.2	38 mM Tx100	0.5 mM TBAA	4.64	303.2 ± 21.06	1.20
			0.77	364.6 ± 19.72	
4.3	38 mM Tx100	1 mM TBAA	4.64	426.4 ± 31.67	1.02
			0.77	434.3 ± 14.29	
4.4	17 mM SDS	0	4.64	381.0 ± 16.17	1.04
			0.77	394.9 ± 10.82	
4.5	17 mM SDS	0.5 mM TBAA	4.64	561.2 ± 36.40	1.06
			0.77	597.0 ± 33.46	
4.6	17 mM SDS	1 mM TBAA	4.64	617.7 ± 28.63	1.04
			0.77	643.4 ± 2.25	

## Conclusions

In this study, the influence of surfactants and halogenide on the dose–response of the DHR123 radio fluorogenic nano clay gel dosimeter was investigated. A good linear dose–response was observed within the dose range of 0–4 Gy in all the prepared conditions except for CTAB. It was found that changing the surfactant sensitizes the dosimeter up to the maximum 8 times (in case of addition of 17 mM SDS). By changing the halide, the sensitivity of the dosimeter is sensitized up to the maximum of 1.95 times (in case of addition of 1 mM TBAA). The addition of surfactant and halide increased the sensitivity up to 16.2 times (in case of addition of 1 mM TBAA and 17 mM SDS). Under this condition, it can be used without dose rate dependence within the range of 0.77 and 4.67 Gy min^−1^. This gel dosimeter is the most sensitive gel dosimeter capable of measuring absorbed dose in the order of 0.01 Gy. This dosimeter is particularly useful for verifying the complex dose distributions in tissue-equivalent materials that are not suitable for pinpoint physical dosimeters. For example, we have reported the application to brachytherapy which has a dose distribution having a steep dose gradient by using normal DHR123 fluorescent gel dosimeter without sensitizer.^44^ Despite the problem of establishing a 2 or 3-dimensional measurement method that maintains the highly sensitive characteristics of this gel dosimeter, it is expected that further applied research on the measuring medical radiation doses and its distribution will proceed.

## Conflicts of interest

There are no conflicts to declare.

## Supplementary Material

RA-010-D0RA02717K-s001
