# Supplementary material for: Sensitivity enhancement of DHR123 radio-fluorogenic nanoclay gel dosimeter by incorporating surfactants and halogenides
Source: RSC Adv. 2020 Aug 4;10(48):28798–806. doi: 10.1039/d0ra02717k (PMC9055799; doi:10.1039/d0ra02717k)
Supplement: RA-010-D0RA02717K-s001 [file RA-010-D0RA02717K-s001.pdf]

Electronic Supplementary Material (ESI) for RSC Advances.
This journal is © The Royal Society of Chemistry 2020

Appendix: Supplementary data

Sensitivity enhancement of DHR123 radio-fluorogenic nano clay gel dosimeter by incorporating surfactants and halogenides.

Anri Mochizuki,[a] Takuya Maeyama,*[a] Yusuke Watanabe,[b] Shinya Mizukami[b]

a. Department of Chemistry, School of Science, Kitasato University, 1-15-1 Kitasato, Minami, Sagamihara, Kanagawa, 252-0373, Japan

b. School of Allied Health Sciences, Kitasato University, 1-15-1 Kitasato, Minami, Sagamihara, Kanagawa, 252-0373, Japan

Normal

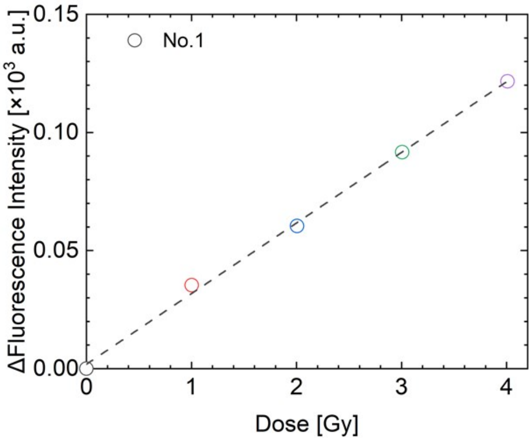

Figure S1. Dose dependence of Δfluorescence intensity obtained from 100uM DHR and 2.5 wt% clay. The dashed lines represent linear fitting.

Tx100

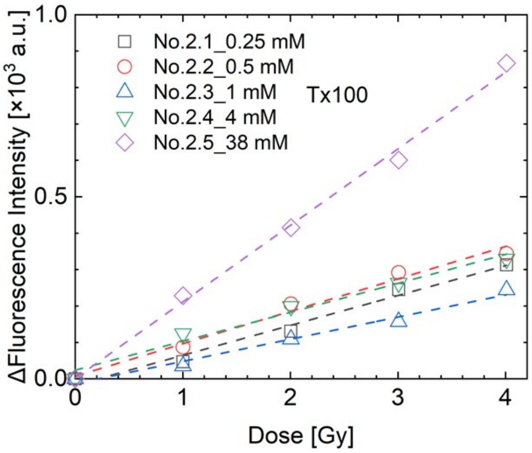

Figure S2. Dose dependence of Δfluorescence intensity: influence of Tx100 concentration.

SDS

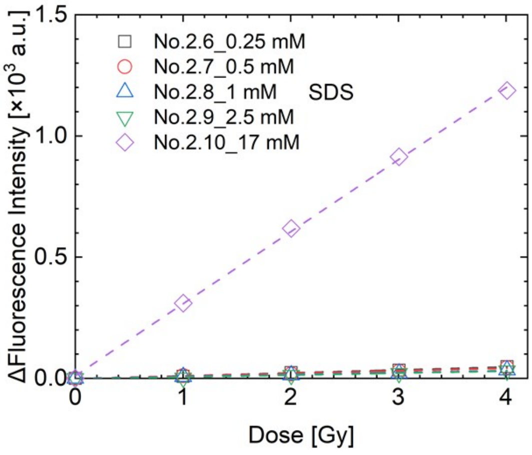

Figure S3. Dose dependence of Δfluorescence intensity: influence of SDS concentration.

TCAA

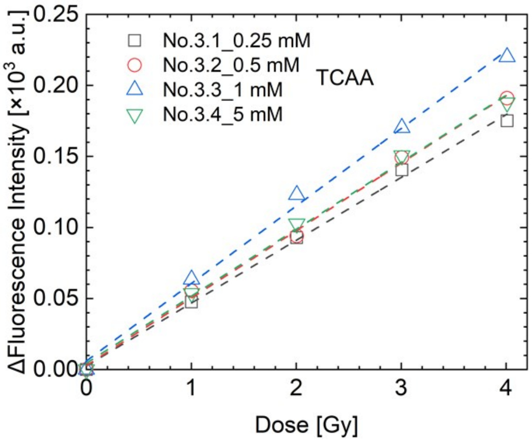

Figure S4. Dose dependence of Δfluorescence intensity: influence of TCAA concentration.

TBAA

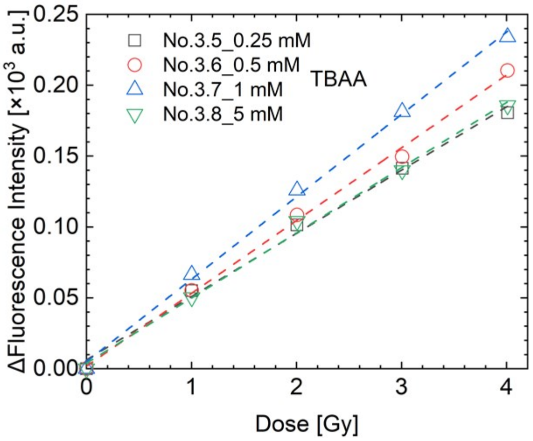

Figure S5. Dose dependence of Δfluorescence intensity: influence of TBAA concentration.

TCE

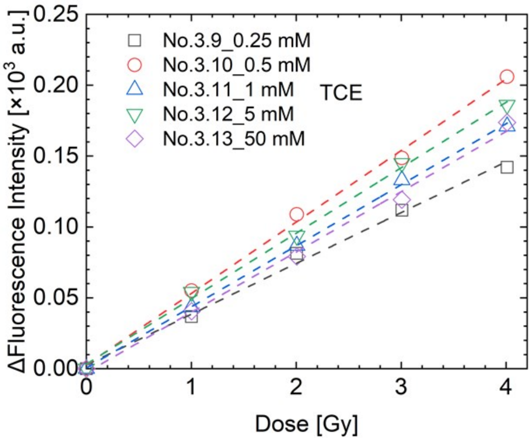

Figure S6. Dose dependence of Δfluorescence intensity: influence of TCE concentration.

38 mM Tx100 and TBAA

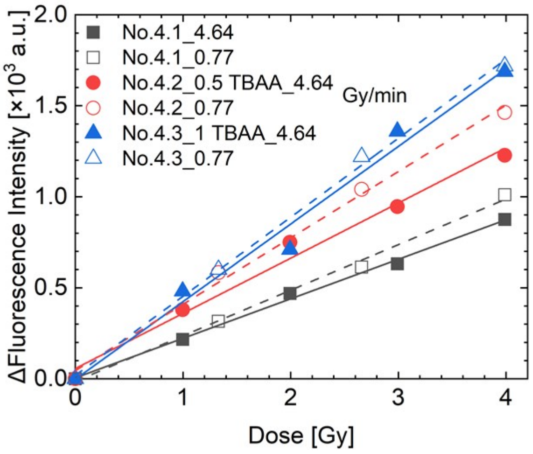

Figure S7. Dose dependence of Δfluorescence intensity: influence of TBAA and 38 mM Tx100 concentration.

17 mM SDS and TBAA

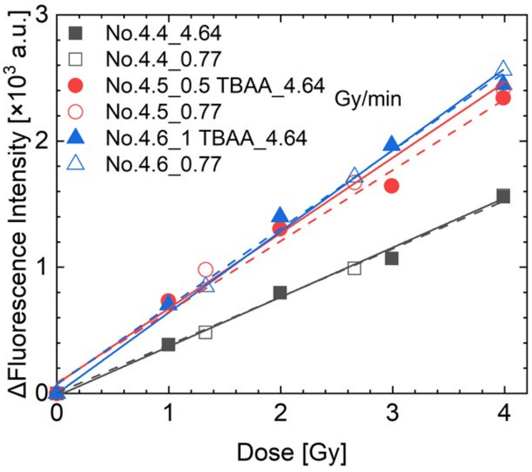

Figure S8. Dose dependence of Δfluorescence intensity: influence of TBAA and 17 mM SDS concentration.

The obtained fluorescence spectrum (solid line) and excitation spectrum (dotted line) are shown below. For the base sample, in order to take into account the effect of the increase in fluorescence intensity over time, measurements were taken at the beginning and at the end of the measurement of the set of samples, and the average value was used as the base value.

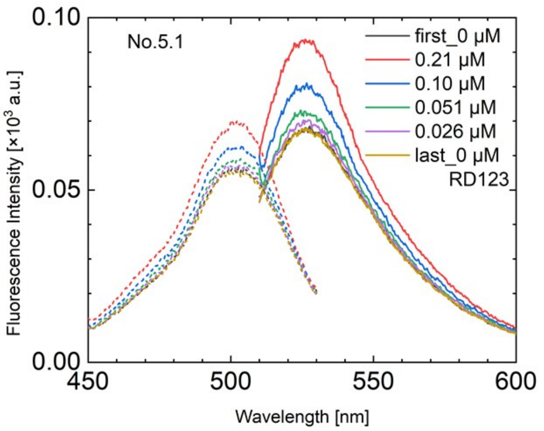

Figure S9. Fluorescence properties of 0 – 0.21 μM RD123 in 100 μM DHR123 in 2.5 wt% Clay. Dashed and solid lines show the excitation and fluorescence spectra, respectively.

Calibration curve for normal condition

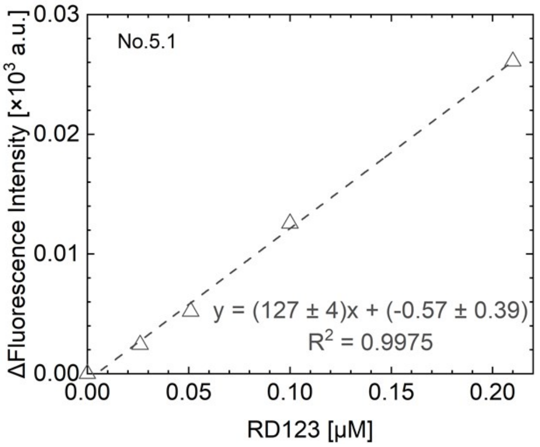

Figure S10. Δfluorescence intensity as a function of RD123 concentration. The dashed lines represent linear fitting.

Calibration curve for TBAA addition

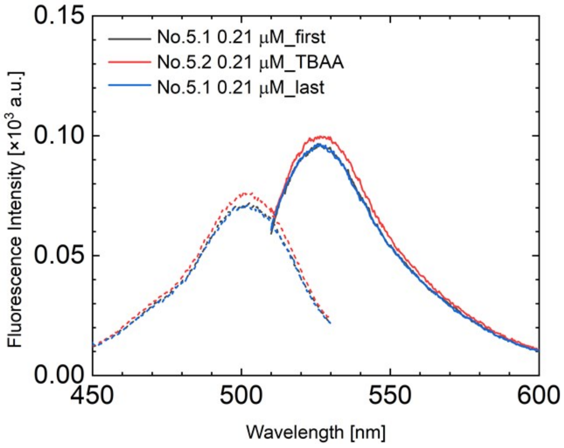

Figure S11. Fluorescence properties of 0.21 μM RD123 in 100 μM DHR123 in 2.5 wt% Clay. Dashed and solid lines show the excitation and fluorescence spectra, respectively.

Calibration curve for Tx100 addition

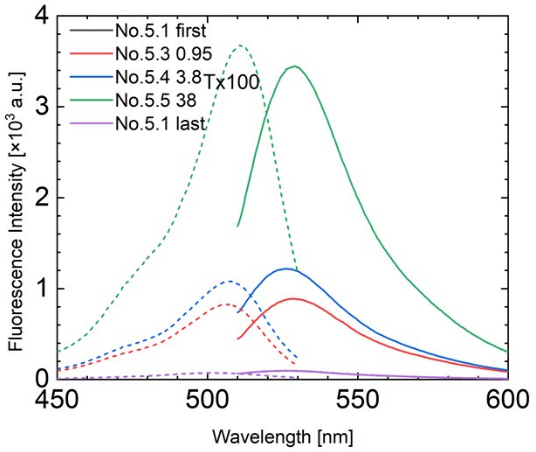

Figure S12. Fluorescence properties of 0.21 μM RD123 in 100 μM DHR123 in 2.5 wt% Clay with Tx100. Dashed and solid lines show the excitation and fluorescence spectra, respectively.

Calibration curve for SDS addition

(a)

Enlarged view

(b)

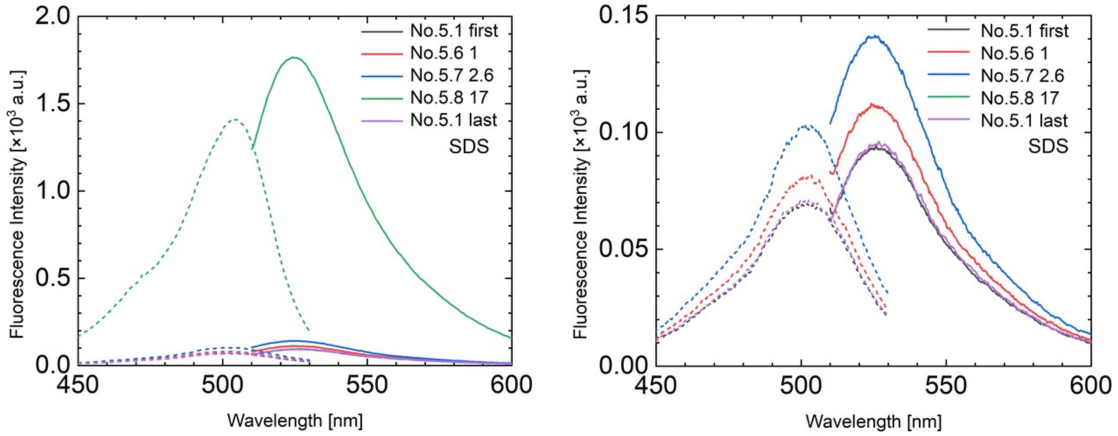

Figure S13. (a) Fluorescence properties of 0.21 μM RD123 in 100 μM DHR123 in 2.5 wt% Clay with SDS. Dashed and solid lines show the excitation and fluorescence spectra, respectively. (b) its enlarged view

Table S1. Dose sensitivity and initial fluorescence intensity of samples prepared with several surfactant

□	surfactant	Conc. [mM]	Transparency	Initial Fluorescence Intensity	Dose sensitivity			Sensitivity Ratio		
1					29.88	±	0.76	1	±	0.03
2.1	Tx100	0.25	clear	782.4	82.13	±	6.12	2.75	±	0.20
2.2	Tx100	0.5	clear	981.4	89.07	±	6.44	2.98	±	0.22
2.3	Tx100	1	clear	1218	60.86	±	4.74	2.04	±	0.16
2.4	Tx100	4	clear	1438	79.51	±	6.87	2.66	±	0.23
2.5	Tx100	38	clear	3117	210.21	±	7.92	7.03	±	0.26
2.6	SDS	0.25	clear	101.8	12.19	±	0.63	0.41	±	0.02
2.7	SDS	0.5	clear	101.6	10.54	±	0.77	0.35	±	0.03
2.8	SDS	1	clear	112.1	8.25	±	0.73	0.28	±	0.02
2.9	SDS	2.5	clear	142.6	8.27	±	0.90	0.28	±	0.03
2.10	SDS	17	clear	2616	297.11	±	4.36	9.94	±	0.15
2.11	CTAB	0.5	very turbid	□	□	□	□	□	□	□

Table S2. Dose sensitivity and initial fluorescence intensity of samples prepared with several halogenide

□	halogenide	Conc. [mM]	Initial Fluorescence Intensity	Dose sensitivity [a.u.]	Sensitivity Ratio
1				29.88 ± 0.76	1.00 ± 0.03
3.1	TCAA	0.25	89.9	44.21 ± 1.37	1.48 ± 0.05
3.2	TCAA	0.5	85.15	47.47 ± 1.49	1.59 ± 0.05
3.3	TCAA	1	83.33	54.54 ± 2.03	1.82 ± 0.07
3.4	TCAA	5	82.82	47.14 ± 1.71	1.58 ± 0.06
3.5	TBAA	0.25	88.05	44.61 ± 1.92	1.49 ± 0.06
3.6	TBAA	0.5	90.6	51.36 ± 1.55	1.72 ± 0.05
3.7	TBAA	1	82.67	58.16 ± 1.54	1.95 ± 0.05
3.8	TBAA	5	104.3	45.99 ± 1.74	1.54 ± 0.06
3.9	TCE	0.25	102.5	35.84 ± 1.61	1.20 ± 0.05
3.10	TCE	0.5	135.2	50.41 ± 1.52	1.69 ± 0.05
3.11	TCE	1	124.3	43.06 ± 0.70	1.44 ± 0.02
3.12	TCE	5	157.5	46.18 ± 1.29	1.55 ± 0.04
3.13	TCE	50	130.5	42.51 ± 1.73	1.42 ± 0.06

Table S3. Dose sensitivity and initial fluorescence intensity of samples prepared by addition of the both a surfactant and halogenide (Dose rate 4.64 Gy/min)

No.	Surfactant	halogenide	Initial Fluorescence Intensity	Dose sensitivity [a.u.]			Sensitivity Ratio		
1	0	0	132.2	38.23	±	1.78	1	±	0.05
4.1	38 mM Tx100	0	3280	217.1	±	7.15	5.68	±	0.19
4.2	38 mM Tx100	0.5 mM TBAA	3381	303.2	±	21.1	7.93	±	0.55
4.3	38 mM Tx100	1 mM TBAA	3307	426.4	±	31.7	11.2	±	0.83
4.4	17 mM SDS	0	3023	381.0	±	16.2	9.97	±	0.42
4.5	17 mM SDS	0.5 mM TBAA	3015	561.2	±	36.4	14.7	±	0.95
4.6	17 mM SDS	1 mM TBAA	2901	617.7	±	28.6	16.2	±	0.75
